# Cardiac Monitoring in Patients with Syncope: Making that Elusive Diagnosis

**DOI:** 10.2174/1573403X10666140214120056

**Published:** 2013-11

**Authors:** Rajesh Subbiah, Pow-Li Chia, Lorne J. Gula, George J. Klein, Allan C. Skanes, Raymond Yee, Andrew D. Krahn

**Affiliations:** 1 Department of Cardiology, St Vincent’s Hospital, UNSW, Darlinghurst, Sydney, NSW, Australia, 2010;; 2 Division of Cardiology, University of Western Ontario, London, Ontario, Canada

**Keywords:** Syncope, loop recorder, holter monitor, cardiac monitor.

## Abstract

Elucidating the cause of syncope is often a diagnostic challenge. At present, there is a myriad of ambulatory cardiac monitoring modalities available for recording cardiac rhythm during spontaneous symptoms. We provide a comprehensive review of these devices and discuss strategies on how to reach the elusive diagnosis based on current evidencebased recommendations.

## INTRODUCTION

Syncope is defined as a transient loss of consciousness secondary to transient global reduction in cerebral blood flow characterized by rapid onset, short duration and spontaneous complete recovery [1]. Syncope is a frequently encountered clinical conundrum with an estimated lifetime prevalence of up to 35% [2]. Syncope accounts for up to 3% of emergency department consultations and 6% of hospital admissions [3-5]. Although the diagnosis may be evident in a minor number of classic presentations, delineating the underlying cause of unexplained syncope can pose a clinical challenge, which is difficult yet worthwhile, as identification of underlying cardiac disease in patients with syncope is associated with higher rates of mortality and morbidity [6]. The most important aspect of the diagnostic challenge is to obtain a comprehensive history and physical examination [7, 8]. The ideal but often unattainable test for elucidating a cause is obtaining comprehensive physiologic data during spontaneous symptoms. Short of that goal, establishing an accurate symptom–rhythm correlation can often provide a diagnosis, making ambulatory outpatient monitoring a powerful diagnostic tool for the evaluation of cardiac arrhythmias. Evolving technologies have provided a wide array of monitoring options for patients suspected of having cardiac arrhythmias, with each modality differing in duration of monitoring, quality of recording, convenience, and invasiveness. 

## HOLTER MONITORING

A standard ECG should be ordered for all patients with syncope [3-9]. Short term ECG monitoring via 3 or, in some cases, 12 surface electrodes is the most common initial investigation in patients who present with syncope or palpitations. Typically this occurs in the emergency room or primary care setting with telemetry and continuous monitoring. More recently, however, wireless telemetry offers the possibility of reviewing continuous ECG recordings instantaneously at particular access points [10]. 

The findings on ECG monitoring must be correlated with symptoms, as heart rate, and even cardiac rhythm, is often uninformative in the absence of clinical correlation. Presyncope is a more common event during ambulatory monitoring but is less likely to be associated with an arrhythmia [11, 12]. Additionally, the ubiquitous nature of presyncope makes it a relatively poor surrogate for the assessment of syncope. 

**Fig. (1):**
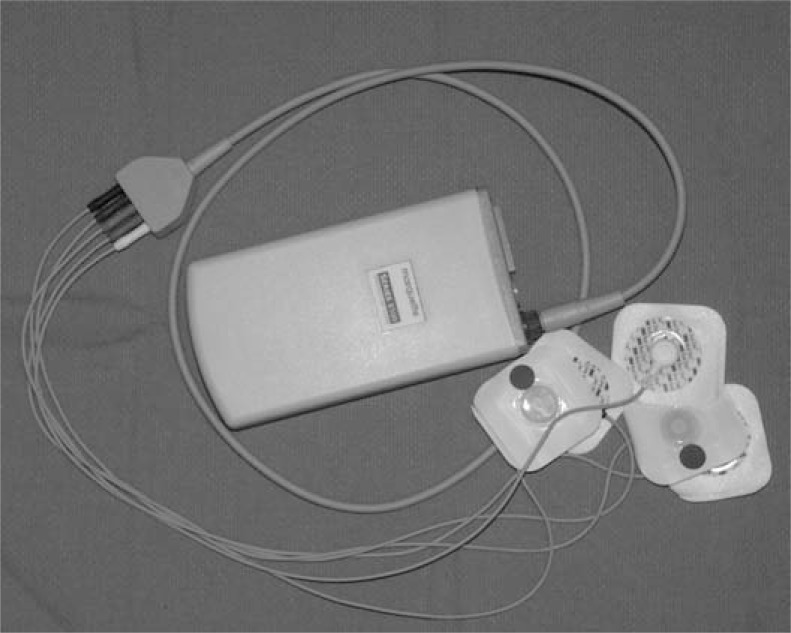
Holter Monitor. The recording device is worn by the patient using a shoulder strap or belt loop, attached to 3-5 skin electrodes for continuous monitoring. An event button (not shown) at the top of the housing of the device is pressed in the event of symptoms to mark the recording. See text for discussion.

The Holter monitor is a portable battery-operated device that connects to the patient using bipolar electrodes, providing recordings from up to 12 ECG leads. Data are stored in the device using analog or digital storage media. The data are transformed into a digital format and analyzed using interpretive software. Additional markers for patient-activated events and time correlates are included, along with a patient event diary, to allow greater diagnostic accuracy. Continuous ECG monitoring is possible for a maximum of 72 hours (See Fig. **1**). This allows the documentation of cardiac rhythm during symptomatic and/or asymptomatic events. Holter monitoring is useful if the clinical history is suggestive of an arrhythmic etiology and the symptoms are frequent enough to be detected within the period of monitoring.

There are, however, a number of disadvantages to Holter monitoring. The major limitation is that patients may not experience symptoms or cardiac arrhythmias during the recording period. The physical size of the device may impair the ability of patients to sleep comfortably or engage in activities that precipitate or reproduce symptoms. Patients are further inconvenienced because the device has to be removed while bathing. There is also considerable variability in patient documentation and recollection of activated events, thereby compromising accurate symptom-rhythm correlation. 

It is therefore not surprising that Holter monitoring has a low diagnostic yield. In several large series of patients undergoing 12 or more hours of ambulatory monitoring for investigation of syncope, only 4% had recurrence of symptoms during monitoring [9, 13, 14]. The overall diagnostic yield of Holter monitoring was 19%. Uncommon asymptomatic arrhythmias such as prolonged sinus pauses, atrio-ventricular block (such as Mobitz type II block), and non-sustained ventricular tachycardia can provide important clues to the diagnosis, often leading to further investigations to rule out structural heart disease and other precipitating factors. While these observations require prompt attention, it is important to interpret the results in the clinical context of the syncopal presentation so that common causes of syncope, such as neurocardiogenic syncope, are not unduly excluded. An example would be nocturnal pauses in a patient with sleep apnea, easily mistaken for intrinsic sinus node disease as a cause of syncope.

It is also important to recognize that normal ambulatory ECG monitoring does not exclude an arrhythmic cause for syncope. Risk stratification scores such as the EGSYS score can be used to estimate the pre-test probability for cardiac syncope [15]. If the pre-test probability is high for an arrhythmic cause, then further investigations such as prolonged monitoring or cardiac electrophysiological studies are required. In a study which evaluated extension of Holter monitoring duration to 72 h [13], there was an increase in the number of asymptomatic arrhythmias detected, but not the overall diagnostic yield. 

## EXTERNAL EVENT RECORDERS 

External event recorders are external devices attached to patients via one to three electrodes with the ability to provide a longer period of monitoring than the standard Holter monitor. They may be patient activated or triggered automatically. The 3 main types of external event recorders are transtelephonic monitors, external cardiac loop recorders and mobile automated cardiac outpatient telemetry (MCOT) monitors.

**Fig. (2):**
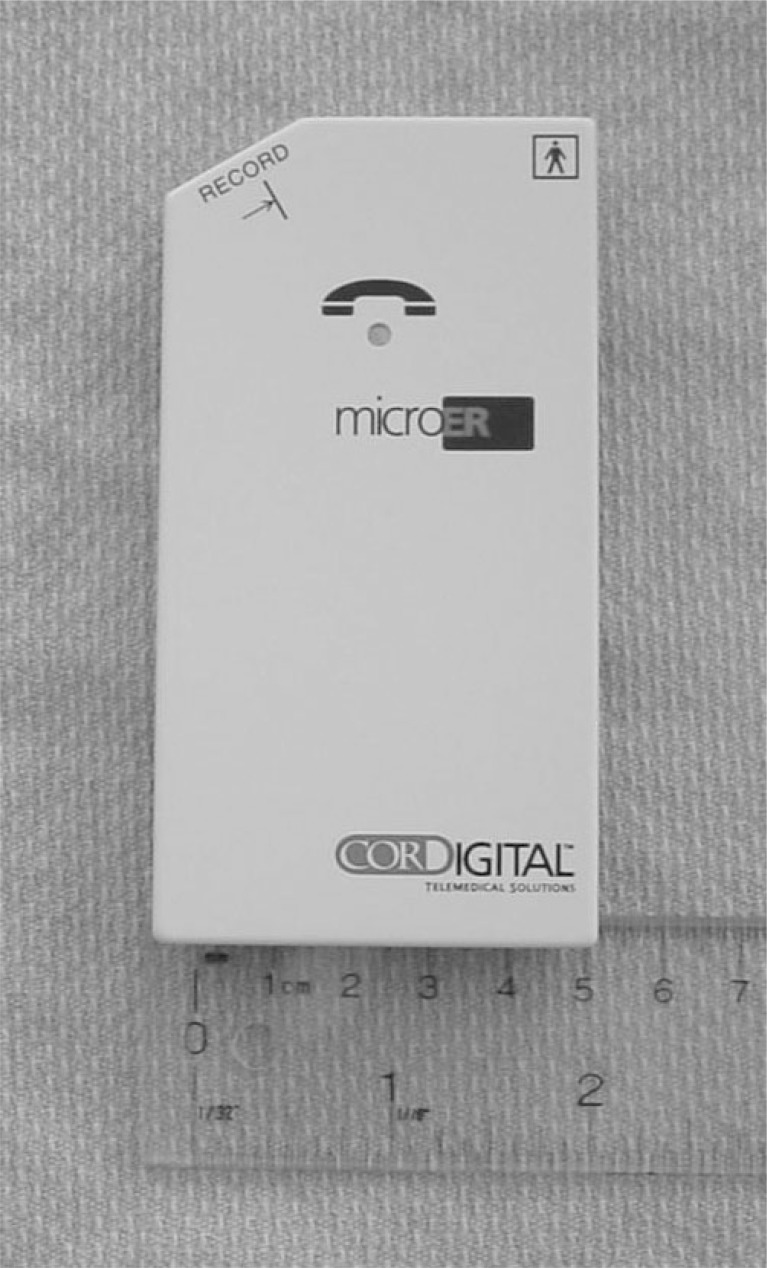
Transtelephonic Monitors. The device is lightweight and portable. Four recording electrodes are present on the back of the device to permit single lead rhythm strip capture. A record button (top left) is pressed at the onset of symptoms, and the recorded event is transmitted to a base station over an analog phone line.

Transtelephonic ECG monitors are recording devices that transmit data via an analog phone line to a base station (Fig. **2**). The signal is then converted to an interpretable recording that is displayed or printed as a single lead rhythm strip. The ECG signals are collected on a real-time 1-2 minute loop. 

**Fig. (3):**
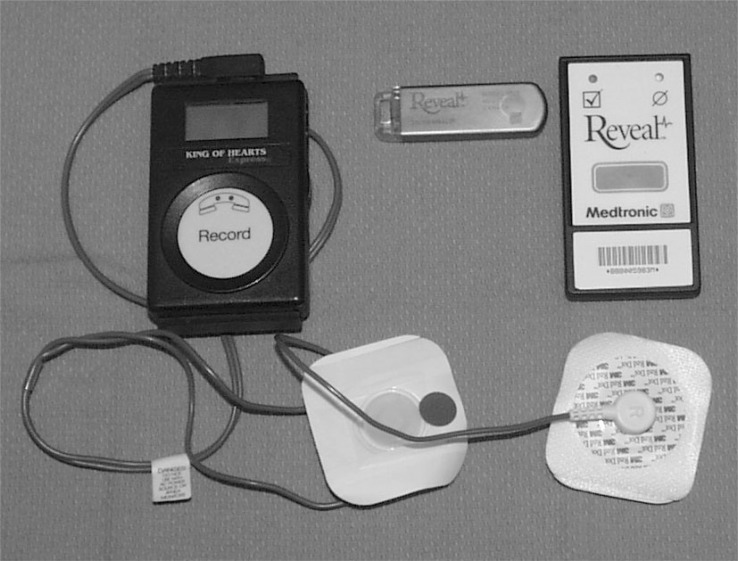
Loop Recorders. An external loop recorder (left) with cables that attach to the patient. The record button is pressed in the event of symptoms to store the previous 9 minutes, and the ensuing minute. The phone receiver is also placed over this button to transmit data over an analog phone line. An implantable cardiac monitor (center) and patient activator (right). The patient activator is used to “freeze” symptomatic events that are retrieved with a pacemaker programmer. Automatic events can also be captured (see text for discussion).

**Fig. (4):**
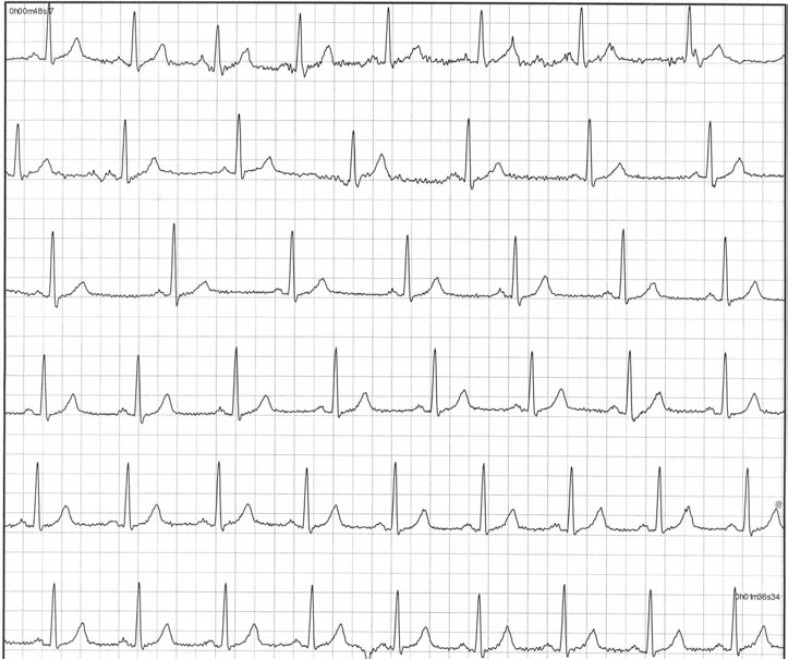
External Loop Recorder Tracing. Sinus rhythm during presyncope is recorded in a 43-year-old female with recurrent unexplained syncope and presyncope. The fluctuation in heart rate is suggestive of neurocardiogenic syncope.

An external cardiac loop recorder continuously records and stores an external single modified limb lead electrogram with a 4-18 minute memory buffer (Fig. **3**, left). After the onset of spontaneous symptoms the patient activates the device, which stores the previous 3-14 minutes, and the following 1-4 minutes, of recorded information. The captured rhythm strip can subsequently be uploaded and analyzed (Fig. **4**) and often provides critical information regarding the onset of the arrhythmia. This system can be used for weeks to months provided weekly battery changes are performed. The recording device is attached with two leads to the patient’s chest wall and needs to be removed for bathing, and can be uncomfortable during sleep. To allow detection of asymptomatic arrhythmias, external loop recorders with an automatic trigger algorithm have been introduced.

MCOT is the most recent advancement in external ambulatory arrhythmia monitoring [16]. Patients wear two to three chest leads attached to a portable sensor that continuously records rhythm strips and transmits the ECG data of pre-specified arrhythmias in real-time to a communication hub at the patient’s home. If the algorithms in the hub detect a significant arrhythmia in keeping with previously designated physician thresholds or if the patient activates the sensor to report symptoms, the monitor automatically transmits the patient’s ECG data to the central station using wireless communications. The data may be screened 24 hours a day by central monitoring station technicians, with potential immediate or deferred referral to the attending physician for evaluation of symptoms, rate and/or rhythm changes. The major drawback of this modality is patient compliance to wearing the device. 

Linzer *et al.* reported the use of patient-activated loop recorders in 57 patients with syncope and non-diagnostic findings on history, physical examination and 24 hour Holter monitoring [17, 18]. A diagnosis was obtained in 14 of 32 patients who had recurrence of symptoms. In the remaining 18 patients, device malfunction, patient non-compliance or inability to activate the recorder was responsible for the lack of diagnosis. Other studies have also reported similar findings [18, 19] and demonstrated that loop recorders are complementary to 24 hour ambulatory electrocardiographic monitoring. The diagnostic yield for external loop recorders in these three studies [17-19] ranged from 24%-47%, with highest yield in patients with palpitations.

A prospective randomized clinical trial compared the utility of external loop recorders to conventional Holter monitoring in a community based referral population with syncope and presyncope [20]. Not surprisingly, the ability to obtain a symptom-rhythm correlation was 22% for Holter monitoring and 56% for the external loop recorder (p < 0.001), with duration of monitoring of 48 hours and 4 weeks, respectively. A higher diagnostic yield was also obtained among patients randomized to Holter monitoring who remained undiagnosed and crossed-over to use of a loop recorder. This trial suggests that loop recorders should be considered as first line monitoring when attempting to establish a symptom rhythm correlation in the initial workup of patients with syncope, unless symptoms are very frequent, or a rhythm sample of 24-48 hours is sought. Twenty-four percent of loop recorder patients failed to activate the device properly, suggesting limited usefulness in some patients [20]. Analysis of factors pertaining to use of external loop recorders has revealed a particularly low diagnostic yield among patients who are unfamiliar with technology, live alone, or have low motivation for achieving a diagnosis [21]. Reiffel *et al*. [22] retrospectively compared the results obtained by Holter monitoring, loop recording and auto-triggered loop recording in 600 patients from a database of approximately 100,000 patients. The auto-triggered loop recording approach provided a higher yield of diagnostic events (36%) compared to loop recording (17%) and Holter monitoring (6.2%). 

External event recorders appear to have the greatest role in motivated patients with frequent spontaneous symptoms that are likely to recur within 4-6 weeks. Given that they are non-invasive and cost effective, they should be considered in all patients in whom an arrhythmic cause for syncope is suspected, keeping in mind that long-term compliance with these devices can be challenging because of electrode and skin-related problems and waning of patient motivation in the absence of recurrent symptoms.

## IMPLANTABLE CARDIAC MONITORS

The implantable cardiac monitor (ICM) has become the investigative tool of choice in recurrent unexplained syncope following negative initial investigations. The ICM permits prolonged monitoring without external electrodes and is ideally suited to patients with infrequent recurrent syncope thought to be due to an arrhythmic cause. Similar to the external event recorders, it is designed to correlate physiology with recorded cardiac rhythms, but is implanted and therefore devoid of surface electrodes and accompanying compliance issues. The ICM also allows for monitoring over much longer time periods than an external event recorder. Commonly available ICMs include the Medtronic Reveal^**® **^and the St Jude Medical Confirm^TM ^series. A typical ICM (Medtronic Reveal DX Model 9528) has a pair of sensing electrodes with 4-cm spacing on a small elongated recording device 6.2 cm long, 1.9 cm wide, and 0.8 cm thick, weighing 15 g (Fig. **3**, center). The projected battery longevity is 36 months. The device can be implanted subcutaneously in the left chest wall with local anesthesia and antibiotic prophylaxis.

Prior to implantation, cutaneous mapping should be performed to optimize the sensed signal and avoid T-wave over-sensing, which can be falsely interpreted as a high rate episode. An adequate signal can usually be obtained anywhere in the left hemithorax [23]. Grubb *et al.* [24] described an anatomic-based approach to ICM placement in 63 patients that did not require cutaneous mapping. Each underwent implantation of ILR in the left upper chest area midway between the supraclavicular notch and left breast area. In all patients, adequate electrocardiographic tracings were obtained at implant without need for preoperative cutaneous mapping. The mean P wave amplitude was 0.12 ± 0.20 mV at implant and at follow-up (6-14 months post-implant), the amplitude was 0.11 ± 0.19 mV. The peak-to-peak QRS amplitude was 0.48 ± 0.15 mV at implant and 0.44 ± 0.16 mV at a follow-up of 6-14 months. This strategy has not been validated.

The recorded bipolar signal is stored in the device as 42 minutes of compressed signal. A compressed signal maximizes memory capability with only marginal loss of quality. The patient, along with a spouse, family member or friend is instructed in the use of the activator at the time of implant. Once an episode is recorded (i.e. a presyncopal or syncopal event occurs) the memory is “frozen” by the patient or a relative using a non-magnetic hand held activator (Fig. **3**, right). The episode is then uploaded for interrogation to a pacemaker programmer. Although heart rate is usually easily ascertained, p waves can occasionally be challenging to interpret. The most recent version of the ICM has programmable automatic detection of tachycardia-bradycardia arrhythmias, pauses and allows for comprehensive remote monitoring without an office visit. The Medtronic CareLink^®^ Home Monitor allows patients to transmit data from their Medtronic Reveal^**® **^ICMs over a standard phone line for review by their physicians. The St Jude Medical Confirm^TM ^ICMs also has transtelephonic monitoring capability, enabling transmission of timely and accurate data. These features enhance the utility of ICMs, especially if patients have frequent saved events or live in remote areas where travel to a dedicated clinic is time consuming and costly. 

**Fig. (5):**
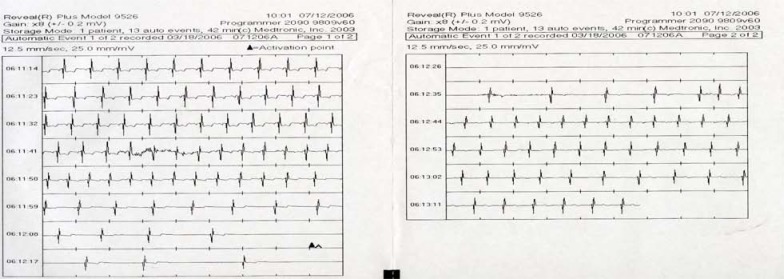
Automatic Event Detection from an ICM. This is a typical tracing of an event captured by an ICM during syncope in a patient. The arrow and letter A denotes automatic activation when the device detects a 3 second pause. Each line constitutes 10 seconds of a single lead rhythm strip. Note the slowing of the sinus rate prior to onset of a prolonged pause, which resulted in syncope. This is consistent with the diagnosis of neurocardiogenic syncope (ISSUE classification 1A).

**Fig. (6):**
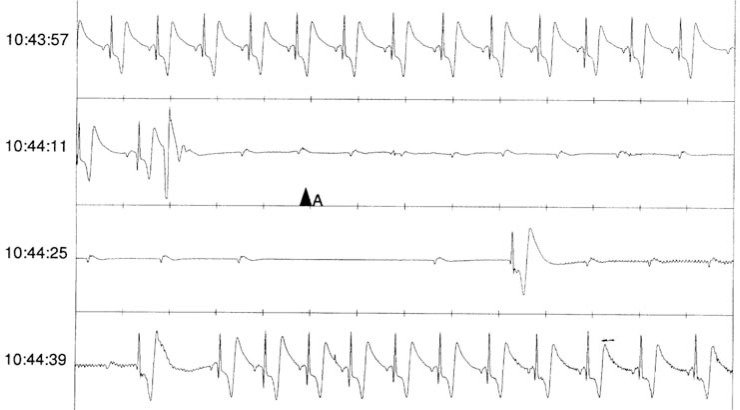
Manual Event Detection from an ICM. Manual activation during presyncope in a 73-year-old male with two previous episodes of unexplained syncope. Note that the sinus rate and PR interval are unchanged surrounding the period of 2:1 AV conduction. This is classified as a 1C response by the proposed ISSUE classification, suggesting intrinsic AV node disease.

A classification system for recorded events has been proposed by Brignole *et al. *[25] (Table **1**) that categorizes the probable mechanism of syncope according to the pattern of bradycardia recorded during spontaneous syncope. An example of the cardioinhibitory component of neurocardiogenic or vasovagal syncope is illustrated in (Fig. **5**). This would be considered a 1A response. Figure **6** illustrates a primary bradycardia (1C response), highly suggestive of intrinsic AV node disease. This classification is useful for research purposes for event classification, and is useful in directing therapy once validated.

Currently there are several studies establishing the utility of ICM in the diagnosis of syncope [25-30]. One of these studies is a multi-centre study of 206 patients [29]. The majority of patients had undergone non-invasive and invasive testing including head-up tilt testing and electrophysiological studies. The etiology of syncope was arrhythmic in 22% of patients [29]. Bradycardia was the most commonly detected arrhythmia (17% vs. 6% tachycardia), usually leading to pacemaker implantation [29]. 

In a group of patients with ongoing seizures despite anticonvulsant therapy, Zaidi *et al.* performed cardiac assessment including head-up tilt testing and carotid sinus massage in all patients, and implantation of an ICM in ten patients [31, 32]. Two of the 10 patients with an ICM had marked bradycardia preceding a seizure; one due to sinus pauses and the other due to heart block. Importantly, this study suggested seizures that are atypical in presentation may have a cardiovascular cause in as many as 42% of cases, and cardiovascular assessment including long term cardiac monitoring with an ICM may play a role in select patients with atypical seizures. 

In three studies [3, 33, 34] from the International Study on Syncope of Uncertain Etiology (ISSUE) investigators, ICMs were implanted in different groups of patients with syncope to assess cardiac rhythm during episodes, after conventional testing. The first study involved tilt tests in 111 patients with unexplained syncope, and ICMs implanted after the tilt test, regardless of result [33]. Syncope recurred in 34% of patients in both the tilt positive and tilt negative group, with marked bradycardia or asystole being the most commonly recorded arrhythmia during follow-up (46% and 62% respectively). The heart rate during tilt testing did not predict spontaneous heart rate response, with a much higher incidence of asystole than expected based on demographics or tilt. This study suggests that observations during tilt testing correlate poorly with cardiac rhythm during spontaneous syncope, and that bradycardia is more common in this population than previously recognized. An example of the cardioinhibitory component of vasodepressor syncope is illustrated in (Fig. **5**).

In the second study, 52 patients with syncope, bundle branch block and negative electrophysiologic testing underwent ICM implantation [3]. Syncope recurred in 22 of the 52 patients with conduction system disease. Long term monitoring demonstrated marked bradycardia mainly attributed to complete AV block in 17, while it excluded AV block in 2. This study confirmed that negative electrophysiologic testing does not exclude intermittent complete AV block, and that prolonged monitoring or consideration of permanent pacing is reasonable in this population.

The third study examined the spontaneous rhythm in 35 patients with syncope, overt heart disease and negative electrophysiologic testing [34]. The underlying heart disease was predominantly ischemic or hypertrophic cardiomyopathy with moderate left ventricular dysfunction. Although previous studies have suggested that patients with negative electrophysiologic testing have a better prognosis, there remains concern regarding risk of ventricular tachycardia in this group. Symptoms recurred in 19 of the 35 patients (54%), with bradycardia in 4, supraventricular tachyarrhythmias in 5 and ventricular tachycardia in only 1 patient. There were no sudden deaths during 16 ±11 months of follow-up.

ISSUE 2 was a prospective, multicenter observational study that investigated the efficacy of therapies based on ICM diagnosis of recurrent suspected neurocardiogenic syncope [35]. The 1-year recurrence rate of syncope in 392 patients was 33%. Among 103 patients with a documented episode, 53 patients were randomized to specific therapy; 47 receiving a pacemaker due to asystole and 6 receiving anti-tachyarrhythmia therapy (catheter ablation: 4, implantable defibrillator: 1, anti-arrhythmic drug: 1). The remaining 50 patients did not receive specific therapy. The 1-year recurrence rate among the 53 patients assigned to a specific therapy was 10% compared with 41% in the patients without specific therapy. The 1-year recurrence rate in patients with pacemakers was 5%. The authors concluded that a strategy based on diagnostic information from early ICM implant, with therapy delayed until documentation of syncope, allows safe, specific, and effective therapy in patients with neurocardiogenic syncope.

PICTURE (Place of Reveal In the Care pathway and Treatment of patients with Unexplained Recurrent Syncope) is the largest prospective, multicenter, observational study to date to evaluate the usage and diagnostic effectiveness of ICMs in the everyday clinical diagnostic work-up of patients with unexplained syncope [36]. Patients were followed up until the first recurrence of syncope leading to a diagnosis or for ≥1 year. In the course of the study, patients were evaluated by an average of 3 different specialists for syncope management and underwent a median of 13 tests (range 9–20). Follow-up visit data were available for 570 subjects. The percentages of patients with recurrence of syncope were 19, 26, and 36% after 3, 6, and 12 months, respectively. Of 218 events within the study, ICM-guided diagnosis was obtained in 170 cases (78%), of which 128 (75%) were cardiac. The results revealed that patients underwent a large number of diagnostic tests before an ICM implant and the use of an ICM was associated with a high diagnostic yield in the overall population with unexplained syncope. Together, these findings imply that if an ICM is implanted early, a reduced number of tests might be needed.

There have been two randomized trials that compared the role of the ICM with a conventional testing strategy for syncope. The Randomized Assessment of Syncope Trial (RAST) [27, 37] was a prospective randomized trial that compared early use of the ICM for prolonged monitoring to conventional testing in patients undergoing a cardiac workup for unexplained syncope. A diagnosis was obtained in 14 of 27 patients randomized to one year ICM monitoring, compared to 6 of 30 undergoing conventional testing with external loop recorder, tilt test and electrophysiology study (52% vs. 20%, p=0.012). Overall, prolonged monitoring was more likely to result in a diagnosis than conventional testing (55% vs. 19%, p=0.0014). Bradycardia was detected in 14 patients undergoing monitoring, compared to 3 patients with conventional testing (40% vs. 8%, p=0.005). These data highlight the diverse etiology of syncope, and also illustrate the limitations of conventional diagnostic techniques. Although there is clear selection bias in enrollment of patients referred to an electrophysiologist for workup, this study suggests that tilt testing has a modest yield when applied to all patients undergoing investigation for unexplained syncope, and that electrophysiologic testing is of very limited utility in patients with preserved left ventricular function. Also, in patients with a negative electrophysiology study for suspected arrhythmia tilt testing has been shown to be of little value in predicting the mechanism of syncope [38].

The other randomized study is the Eastbourne Syncope Assessment Study (EaSyAS) [39]. Two hundred and one patients presenting to a single institution with recurrent syncope without a definite diagnosis following a basic clinical work-up were randomly assigned to ICM implantation (n**=**103) or conventional investigation and management (n**=**98). Over a mean follow-up period of 276 ± 134 days, there were further syncopal events in 43% of the ICM group compared with 33% of the conventional strategy group. Thirty-three patients in the ICM group and four in the conventional strategy group received an electrocardiographic diagnosis (33% vs 4%, HR 8.93, 95% CI 3.17 to 25.2, p<0.0001). Seventeen-month follow-up data from the same group of patients were reported in 2006 [40]. Forty-three per cent of the ICM group and 6% of the conventional testing strategy group received an electrocardiographic diagnosis (HR 6.53, 95% CI 3.73 to 11.4, p<0.0001).

## STRATEGIES FOR CHOOSING PROLONGED MONITORING

Table **2** summarizes the comparative advantages, limitations and indications for the various modes of ambulatory electrocardiographic monitoring. The literature, including recently updated guidelines and position papers from the European Society of Cardiology and European Heart Rhythm Association [1,41], supports the early use of the ICM in an initial phase of the diagnostic work-up of patients with recurrent unexplained syncope. The optimal patient for prolonged monitoring with an external event recorder or ICM has symptoms suspicious for arrhythmia; namely abrupt onset with minimal prodrome, typically brief loss of consciousness and complete resolution of symptoms within seconds to minutes. ISSUE 2 suggested that documentation of the cardioinhibitory component of vasovagal syncope might identify a group of patients that respond well to pacing. Brignole and colleagues addressed this hypothesis, by evaluating the effect of placebo pacing therapy [42]. Syncope recurred in 38% of patients randomized to placebo vs. 34% randomized to no treatment. The recurrence rate with active cardiac pacing was 15%. The authors suggested that the use of specific selection criteria for pacing, such as characteristics of the observed cardioinhibitory reflex may identify those who will respond to cardiac pacing [42]. 

After clinical assessment, including assessment of left ventricular function, a decision must be made if the patient’s underlying condition is potentially life threatening. All reports using the ICM have suggested a low incidence of life-threatening arrhythmia or significant morbidity with a prolonged monitoring strategy. This suggests a good prognosis for patients with recurrent unexplained syncope in the absence of left ventricular dysfunction or with negative electrophysiologic testing. This finding was particularly striking in the negative electrophysiologic testing arm of the ISSUE study (see discussion above).

Lastly, syncope fails to recur during long term monitoring in almost one third of patients even in the presence of frequent episodes prior to ICM implantation. This suggests that the cause of syncope in some instances is self-limiting, reflecting a transient physiologic abnormality.

## CONCLUSIONS

Syncope, although relatively common, remains a significant diagnostic dilemma for clinicians, despite advances in knowledge pertaining to mechanism. A careful history and physical examination are crucial in the course of differentiating syncope from other causes of loss of consciousness. The ultimate diagnostic goal is to correlate symptoms to rhythm disturbances, and accurate attainment of this goal requires the judicious use of monitoring strategies. Ambulatory cardiac monitoring has provided a powerful means to elucidate etiology of presyncope or syncope. The choice of ambulatory monitoring modality is influenced by index of suspicion of cardiac arrhythmias, frequency and nature of symptoms and diagnostic yield of the monitoring device. The clinician should consider early use of ICMs and when an arrhythmia is suspected based on clinical presentation and initial non-invasive testing.

## Figures and Tables

**Table 1.** ISSUE classification of detected rhythm from the ICM.

Classification	Sinus Rate	AV Node	Comment	Presumed Mechansim
Asystole (RR>3 sec)				
1A	Arrest	Normal	Progressive sinus bradycardia with sinus arrest:	vasovagal
1B	Bradycardia	AV block	AV block with associated sinus bradycardia:	vasovagal
1C	Normal or tachycardia	AV block	Abrupt AV block without sinus slowing	intrinsic AV node disease
Bradycardia				
2A	Decrease>30%	Normal		vasovagal
2B	HR<40 for >10 seconds	Normal		vasovagal
Minimal HR change				
3A	<10% variation	Normal	Suggests unlikely vasovagal	non-cardiac cause
3B	HR increase or decrease 10-30%, not <40 or >120 bpm	Normal		vasovagal
Tachycardia				
4A	Progressive tachycardia	Normal	Sinus acceleration typical	orthostatic intolerance or non-cardiac cause
4B	N/A	Normal	Atrial fibrillation	Mixed – may be a component of vasovagal as well
4C	N/A	Normal	Supraventricular tachycardia	
4D	N/A	Normal	Ventricular tachycardia	

HR – heart rate, N/A – not applicable. Adapted from Brignole M, Moya A, Menozzi C, Garcia-Civera R, Sutton R. Proposed electrocardiographic classification of spontaneous syncope documented by an implantable loop recorder. Europace. Jan 2005;7(1):14-18 with permission.

**Table 2.** Comparison of ambulatory electrocardiographic monitoring devices.

	Advantages	Limitations	Indications	Diagnostic yield
Holter monitor	Low cost; Continuous monitoring	Short duration of monitoring with low diagnostic yield	Patients with very frequent symptoms (≥1 per week)	6%-22% [9, 20, 22]
Transtelephonic monitor	Low cost	Poor electrocardiographic recordings; Short lasting arrhythmias are not recorded; Patient activation required; Poor patient compliance to wearing device	Compliant patients with inter-symptom interval ≤ 4 weeks	23%-42% [17-19]
External loop recorder	Retrospective and prospective electrocardiographic records; Possibility to record asymptomatic arrhythmias automatically	Poor electrocardiographic recordings; Poor patient compliance to wearing device; Continual device maintenance required	Compliant patients with inter-symptom interval ≤ 4 weeks	24%-47% [17-19]
Mobile cardiac outpatient telemetry	Continuous monitoring; Patient activation to report symptoms	Poor patient compliance to wearing device; Continual device maintenance required; Cost; Not widely available	Compliant patients with inter-symptom interval ≤ 4 weeks	41%-61% [16]
Implantable cardiac monitor	Prolonged monitoring without external electrodes; Highest diagnostic yield	Invasive implantation with risk of local complications; High cost	Early phase of evaluation of patients with recurrent syncope of uncertain origin who have absence of high-risk criteria that require immediate hospitalization or intensive evaluation and a likely recurrence within device battery longevity	43%-78% [25-30]
